# Assembling Reads Improves Taxonomic Classification of Species

**DOI:** 10.3390/genes11080946

**Published:** 2020-08-17

**Authors:** Quang Tran, Vinhthuy Phan

**Affiliations:** Department of Computer Science, University of Memphis, Memphis, TN 38152, USA; vphan@memphis.edu

**Keywords:** metagenomic classification, short-read sequencing, metagenomic assembly

## Abstract

Most current approach to metagenomic classification employ short next generation sequencing (NGS) reads that are present in metagenomic samples to identify unique genomic regions. NGS reads, however, might not be long enough to differentiate similar genomes. This suggests a potential for using longer reads to improve classification performance. Presently, longer reads tend to have a higher rate of sequencing errors. Thus, given the pros and cons, it remains unclear which types of reads is better for metagenomic classification. We compared two taxonomic classification protocols: a traditional assembly-free protocol and a novel assembly-based protocol. The novel assembly-based protocol consists of assembling short-reads into longer reads, which will be subsequently classified by a traditional taxonomic classifier. We discovered that most classifiers made fewer predictions with longer reads and that they achieved higher classification performance on synthetic metagenomic data. Generally, we observed a significant increase in precision, while having similar recall rates. On real data, we observed similar characteristics that suggest that the classifiers might have similar performance of higher precision with similar recall with longer reads. We have shown a noticeable difference in performance between assembly-based and assembly-free taxonomic classification. This finding strongly suggests that classifying species in metagenomic environments can be achieved with higher overall performance simply by assembling short reads. Further, it also suggests that long-read technologies might be better for species classification.

## 1. Introduction

The species level classification is an important problem in metagenomics analysis. Computational workflows aim for the identification of microbial species that are present in metagenomics samples. Examples include studies that assessed the host-microbe interactions in the gut microbiome to gain better insight into human health [1], revealed ecological differentiation of closely related bacteria [2], uncovered the presence of ancient sub-populations of marine bacteria [3], and highlighted extensive intra-species recombination [4,5].

Methods for classification and profiling of microbial communities are diverse. CLARK [6] uses a database of k-mers that aims to uniquely describes genomic regions of each targeted microbes. GOTTCHA [7] has a different approach to identifying unique genomic regions of targeted microbes by using a combination of empirical data and machine learning methods. Kraken [8] also utilizes k-mers, but builds taxonomic trees that help differentiate closely related microbes. MetaPhlAn2 [9] employs a similar taxonomic approach, but narrows read alignment and its analysis is on a set of only around one million markers.

More recent technologies can produce very long reads, but at the expense of having higher costs and much higher error rates [10]. However, longer reads have been found to be more appropriate or better compared to short reads in certain studies [11]. Single-molecule sequencing (SMS) offers exceptionally long reads that enable direct sequencing of genomic regions that are difficult to sequence with short reads, including long repetitive elements, extreme GC-content regions, and complex gene loci. Similarly, these platforms enable structural variation characterization at previously unparalleled resolution and direct detection of epigenetic marks in native DNA [12]. Similarly, the PacBio sequencing system can capture full-length 16S rRNA sequences [13]. Third-generation nanopore sequencing offers many solutions to the current problems of using whole metagenome sequencing (WMS) for infectious disease diagnostics. It has been successfully utilized for pathogen detection, AMR prediction, and characterization of mixed microbial communities [14].

While long read technologies are more appropriate for certain studies, short read technologies are mature and less expensive. Is it possible to leverage known strengths of short read technologies to garner the high performance of long reads?

In this paper, we demonstrate that it is possible to improve the performance of species classification in metagenomic applications using long reads that are assembled from short reads. This finding has two major implications. First, it suggests that many existing studies that utilize short reads can benefit from long reads that are assembled from the short reads. Although there is an extra computational cost of assembly and minor modification to the existing workflows, the increase in performance might justify the cost. Second, this finding suggests that there are potential gains in utilizing long-reads technologies in this type of applications. As current long-read technologies have different characteristics from short-read technologies in terms of cost and sequencing errors, the trade-offs between these pros and cons remain to be investigated.

## 2. Method

Most metagenomic classifiers, including those that we studied in this paper, consist of two main steps. In the preprocessing step, a classifier utilized reference genomic sequence of existing species to build an index or reference table. The index or reference table was built only once for a metagenomic environment. In the classification step, the classifier used the index or reference table to classify metagenomic data, in the form of reads, and make predictions.

**Figure 1:**
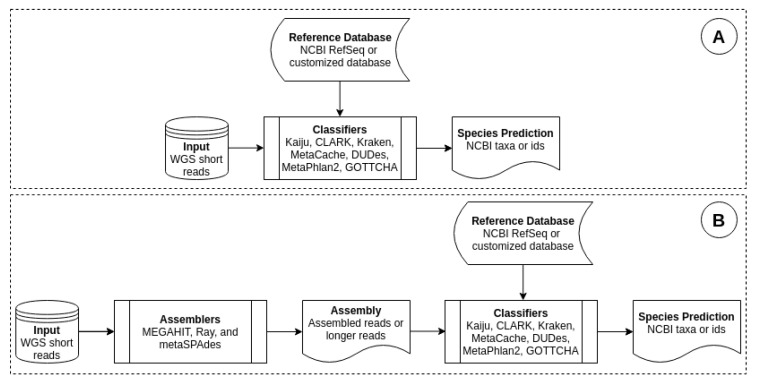
Workflow of metagenomic classification: (**A**) original workflow, which uses short reads, (**B**) modified workflow, which uses assembled reads. Metegenomic classifiers are Kaiju, CLARK, Kraken, MetaCache, MetaPhlAn2, DUDes and GOTTCHA. Metagenomic assemblers are MEGAHIT, metaSPAdes and Ray.

Our method interrupted this workflow by modifying the classification step. Before feeding short reads as inputs to a classifier, we assembled them into long reads. Figure 1 depicts the process of comparing a classifier’s performance on short reads and long reads. Figure 1A is the standard workflow of a classifier, where it took a short reads dataset as input and outputted species that it predicted to be present in the sample. Figure 1B shows a workflow, in which the same short reads were first assembled before feeding to the classifier.

Different methods may have different types of prediction formats, which can be species label for each read, or predicted species for the entire dataset, or predicted percentages of species in the sample (in case of metagenomics profiling). Metagenomic classifiers outputted a rank separated taxanomic profile with relative abundances, whereas binning classifiers provided sequence identification, length used in the assignment, and taxon as output. The classifier’s outputs were then converted into species names, which produced a list of species.

Each classifier required a reference database of genomic sequences to classify metagenomic reads into species. We used complete genomes of bacteria archaea, and viruses from NCBI to construct this database for each classifier. We removed species that were labeled unclassified or unknown because they might cause problems for taxonomic prediction [15].

For consistency, we used the NCBI taxonomy database [16] to standardize results from different classifiers. Further, for classifiers that produced strain-level predictions, we converted them to species-level predictions so that the results could be compared consistently across different classifiers.

We compared the outputs of classifiers using default parameters at the species level because not all classifiers still predicted at strain level. Species is a taxonomic rank more relevant in clinical diagnostics or pathogen identification than genus or phylum. Although some clinical diagnosis and epidemiological tracking often requires identification of strains, genomic databases remain poorly populated below the species level [17]. Evaluation was done in a similar way to [17,18]. For each classifier, we evaluated predicted species produced with assembled reads and predicted species produced with original short reads.

### 2.1. Classifiers

We evaluated with a set of seven metagenomic classifiers: Kaiju (version 1.7.2) [19], CLARK (version 1.2.6) [6], Kraken (version 1.1.1) [8], MetaCache (version 0.6.1) [20], MetaPhlAn2 (version 2.6.0) [9], DUDes (version 0.08) [21], and GOTTCHA (version 1.0c) [7]. The choice was motivated by recent publications comparing the performance of such tools [17].

Kaiju, CLARK, Kraken, MetaCache are k-mer based methods for metagenomic read classification. CLARK and Kraken were run with the default k-mer size of 31, while MetaCache use 16-mers by default. Kaiju was run in the fastest MEM mode (with minimum fragment length m=11), as well as in the heuristic greedy mode (with minimum score s=65).

On the other hand, both MetaPhlAn2 [9] and DUDes [21] have to use results of read-to-reference mapping from Bowtie2 [22]; however, for some longer contigs (several million bps), Bowtie2 (version 2.3.4.2) crahsed. We used a read mapper designed for both short and long reads: Minimap2 (version 2.17) [23] as an alternative for mapping reads to reference genomes.

While running the classifiers above, we specified the “paired-end reads” option for raw read input as well as the “singleton read” option for assembled read input.

### 2.2. Assemblers

MEGAHIT (version 1.2.9) [24], metaSPAdes (version 3.13.1) [25], Ray (version 2.3.1) [26] were used to assemble short-reads into contigs. These tools were selected based on their popularity for assembling metagenomic reads [27].

Assemblers were launched with (mostly) default parameters; taking a pair of FASTQ files that contains raw reads and then producing a single FASTA file that contains assembled reads for each dataset. The file names of assembled reads from MEGAHIT, metaSPAdes, and Ray were “final.contigs", “contigs" and “Contigs" respectively.

Ray parallelized assembly computations using the Message Passing Interface (MPI) standard, a run agent “mpirun". While metaSPAdes consumes very high memory, MEGAHIT specified multiple computational threads and optionally a graphical processing unit for improving its runtime. Due to the scope of this work, we do not report the runtime as well as memory usage of the assemblers.

## 3. Result

### 3.1. Experimental Design

The main hypothesis was that the classification or identification of microbes in metagenomics samples was better done with long reads than with short reads. We aimed to design a controlled experiment to verify the hypothesis. To achieve this, we evaluated the ability to detect species in metagenomics samples of several well-known classifiers on several short-reads datasets and derived long-reads datasets. The choice of which long-reads datasets were used to compare against which short-reads datasets was an important design decision. If we chose a long-reads dataset produced by a current technology to compare against a short-reads dataset produced by a different technology, the result might be due to differences in technologies rather than in read lengths. As our goal was to examine the impact of read lengths on classification, we chose to use long reads that are derived from the same short reads. These derived long-reads datasets were constructed by assembling short reads from the datasets that are used to evaluate the classifiers’ performance. Although this design choice removed the effect of sequencing technologies, it introduced the potential effect of assembling reads on the result. To address this, we evaluated classifiers with different assemblers to remove algorithmic bias on classification performance.

We chose classifiers and assemblers that were free and widely used. We excluded any tools that were difficult to use or install, as well as those that were lacking in support of any kind. We selected seven popular classifiers: Kaiju [19], CLARK [6], Kraken [8], MetaCache [20], MetaPhlAn2 [9], DUDes [21], and GOTTCHA [7] and three metagenomic assemblers: MEGAHIT [24], metaSPAdes [25], Ray [26]. These tools employ different algorithmic techniques.

### 3.2. Performance Assessment

Classifiers were evaluated with synthetic and real samples. Although some tools could work on the strain level, we evaluated classification results at species levels since most methods still do not provide strain level identifications. Classification performance of synthetic data was measured in terms of precision, recall, and F1.

$$Precision=\frac{\mathrm{TP}}{\mathrm{TP}+\mathrm{FP}};Recall=\frac{\mathrm{TP}}{\mathrm{TP}+\mathrm{FN}};F1=\frac{2\cdot precision\cdot recall}{precision+recall}$$

where TP (true positives): the number of correctly classified species; FP (false positives): the number of incorrectly classified species; FN (false negatives): the number of incorrectly classified non-species, by each method.

To access classifiers’ performance on real data, we define the overall pairwise similarity of a method *c* to other methods as

$$\frac{\sum_{i=1,c\neq i}^{n}\left|S_{c}\cap S_{i}\right|}{\sum_{i=i}^{n}\left|S_{c}\cap S_{i}\right|}$$

where, $S_{i}$ is the number of species predicted by method *i*. This similarity is between 0 and 1. The closer it is to 1, the higher the overall similarity to other methods.

### 3.3. Data

We used the Mende datasets [28], which are synthetic data consisting of 890 genomes representing 457 strains, species, and sub-species. The simulators used to generate custom metagenomic data are freely available, as are the datasets we used http://www.bork.embl.de/~mende/simulated_data/.

Mende datasets were simulated for Sanger sequencing, pyrosequencing, and Illumina sequencing. For each technology, three metagenomes were simulated to mimic different community complexities 10 species (10 s), 100 species (100 s), and 400 species (400 s). However, the Sanger sequencing, pyrosequencing technologies seemed obsolete/out-of-date. We tested our hypothesis on Illumina paired-end raw reads of Mende datasets, which is a very widely used sequencing platform.

To test our hypothesis with the real data, we used the gut microbiome data [29]. The metagenomic shotgun-sequencing data for two samples (ERR2017411, ERR2017412) was downloaded from the European Bioinformatics Institute (EBI) database under the accession code ERP023788.

All data used were paired-end reads within the Illumina platform. Synthetic data consists of 26 million reads for each dataset (10 s, 100 s, and 400 s) with the read length of 75 bp. Real data had 17 million reads for each sample with the read length of 90 bp. There Methods a slight difference in read lengths between synthetic data and real data; however, the read lengths from 75 bp to 100 bp were reported [30,31] to produce the same alignment results.

To analyze the distribution of lengths of long reads and assembled contigs, we acquired two sets of metagenomic sequencing data: PacBio RSII data from the Microbial Mock Community B of the Human Microbiome Project (HMP Set 7) and Nanopore GridION sequencing data of the Zymo Community Standards 2 synthetic community (Zymo-GridION-EVEN-BB-SN). The HMP7 data are publicly available (https://github.com/PacificBiosciences/DevNet/wiki/Human_Microbiome_Project_MockB_Shotgun). The Zymo data are publicly available (https://github.com/LomanLab/mockcommunity).

### 3.4. Findings

Using assembled reads, four out of seven classifiers increased their precision by up to 2×, while maintaining similar recall; see Table 1. These four classifiers were Kaiju, CLARK, Kraken and MetaCache. The improvement in performance was most significant for smaller datasets. With the dataset 10 s, which consisted of 10 species, CLARK, for example, benefited from a 50× increase in precision with the same recall, when reads were assembled by any of the three assemblers. With the dataset 100 s, which consisted of 100 species, CLARK benefited from a 3−4× increase in precision with the same recall, when reads were assembled by any of the three assemblers. With the dataset 400 s, which consisted of 400 species, CLARK benefited from a 1.04× increase in precision with the same recall. Similarly, other three classifiers benefited from assembled reads. Kraken and MetaCache benefited from increases in both precision and recall with the larger datasets 100 s and 400 s.

MetaPhlAn2’s performance got worse with assembled reads, compared to its performance on unassembled short reads. We also observed that DUDes and GOTTCHA did not benefit from assembled reads. The overall F1 scores were highest when reads were assembled by MEGAHIT and metaSPAdes.

**Figure 2:**
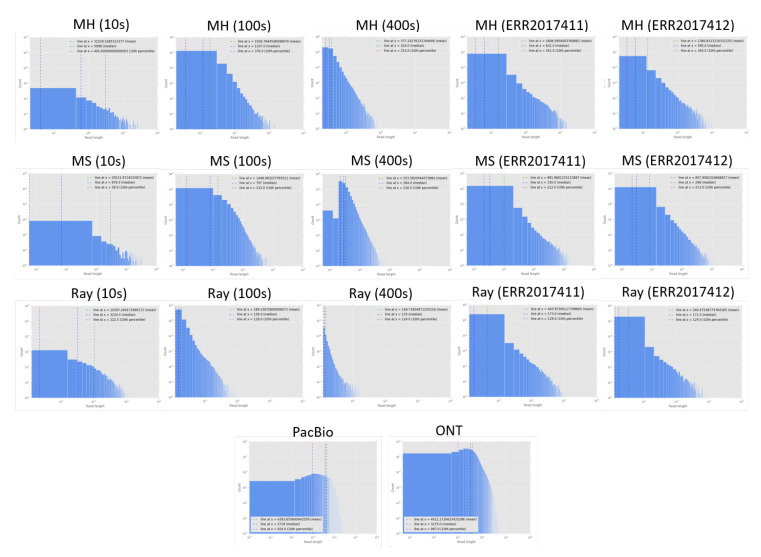
Contig length distribution compared to PacBio and ONT long read length distribution. Contigs were assembled across datasets (from left to right): 10s, 100s, 400s, ERR2017411, ERR2017412 and by different assemblers (from top to bottom): MEGAHIT (MH), metaSPAdes (MS), Ray. The bottom subfigures are PacBio (left) and ONT (right) read length distribution.

We report the assembly statistics (Table 2) and read length distribution of assembled reads compared to two datasets of current long-read technologies (Figure 2). Note that due to highly fragmented reads and contigs, the read/contig length distribution was log scaled. In Table 2, the performance of Ray with MetaPhlAn2, DUDes and GOTTCHA on the 400 s simulation dataset was much worse than that without assembly, likely because the number of contigs on 400 s was significantly smaller than that of 10 s, and 100 s. Therefore, Ray was not a good choice for assembling reads with a high number of species in the sample. We suggest that later assembly algorithm developments should consider the estimate number of species as an option.

We observed that with synthetic data, the majority of classifiers predicted much fewer species when reads were assembled; see Table 3. With fewer predicted species, there should be fewer false positives. Even if prediction mistakes were not random, making fewer positive predictions will naturally decrease the number of false positives, which tends to increase precision. This observation likely explains the drastic observed increase in precision, while maintaining similar recall, across the board with synthetic datasets. With real datasets, we observed a similar behavior that classifiers predicted much fewer species when reads were assembled. Although we could not compute precision and recall with real data, the same trend suggests that as with synthetic data, classifiers should be much more precise when reads were assembled. This increase in precision should, similarly, be drastic when the datasets have much fewer species than the index database that were used by classifiers to classify species.

Additionally, Table 4 shows that with real datasets, the overall pairwise similarity decreased with assembled reads. This suggests that with assembled reads, classifiers had a higher chance of showing their uniqueness in predicting species.

## 4. Discussion

In this work, we showed the promising prospect of utilizing long reads in identifying species in metagenomic samples. Long reads, used in this study, are assembled from the same short reads, which were used to compare classification performance. This was performed to remove potential side effects of different sequencing technologies. As future long-read technologies achieve fewer sequencing errors and become less expensive, their use for species classification in metagenomics should be desirable.

At present, we have demonstrated that we can leverage the advantage of long reads by assembling short reads that would otherwise be used for species classification. We showed that at least two of the currently popular assemblers can be used for this purpose. We observed that MEGAHIT and metaSPAdes produced higher N50s across datasets, while Ray had lower N50s. In fact, it failed to assemble reads when the datasets contained 400 species. A quick comparison between metaSPAdes and MEGAHIT assemblers across all the datasets considered in this study confirmed that metaSPAdes performs better for a smaller dataset (10 s) while MEGAHIT performs better for larger datasets (100 s and 400 s).

We think that Kaiju, CLARK, Kraken, and MetaCache benefited from the longer reads because of their approach of using k-mers as unique markers to distinguish closely related species. On the other hand, MetaPhlAn2, DUDes and GOTTCHA have built-in statistical post-processing procedures that align reads to reference genomes, which appear not benefit from longer reads.

## 5. Conclusions

We demonstrated that improvement in taxonomic classification can be achieved with a novel assembly-based protocol. Specifically, we compared performance of popular metagenomic classifiers on short reads and longer reads, which are assembled from the same short reads. Using a number of popular assemblers to assemble short reads, we discovered that most classifiers made fewer predictions with longer reads and that they achieved higher classification performance on synthetic metagenomic data. Specifically, across most classifiers, we observed a significant increase in precision, while recall remained the same, resulting in higher overall classification performance. On real metagenomic data, we observed a similar trend as in the case of synthetic data that classifiers made fewer predictions. This suggested that they might have the same performance characteristics of having higher precision while maintaining the same recall with longer reads.

This finding has two main implications. First, it suggests that classifying species in metagenomic environments can be achieved with higher overall performance simply by assembling short reads. This finding can make a big impact on the many existing studies that utilize short reads. The modification to their existing workflow is minimal, although there is an extra computational cost of assembling short reads. We showed that a number of existing assemblers could be used for the purpose of assembling short reads into contigs for this specific purpose. Second, this finding suggests that the use of long-read technologies in taxonomic classification might result in significant improvements. Current long-read technologies tend to have higher sequencing errors and are more expensive compared to short-read technologies. The trade-offs between the pros and cons may be further investigated.

## Figures and Tables

**Table 1:** Precision, recall, F-1 of species-level classification of four metagenomic classifiers on three synthetic short read datasets, which are, respectively, not assembled and assembled by three assemblers: MEGAHIT (MH), metaSPAdes (MS), and Ray.

		Kaiju			CLARK			Kraken			MetaCache			MetaPhlAn2			DUDes			GOTTCHA		
		Pre	Rec	F1	Pre	Rec	F1	Pre	Rec	F1	Pre	Rec	F1	Pre	Rec	F1	Pre	Rec	F1	Pre	Rec	F1
10 s	n/a	0.02	1.0	0.04	0.02	1.0	0.05	0.03	1.0	0.06	0.20	1.0	0.33	1.0	1.0	1.0	1.0	0.90	0.94	1.0	1.0	1.0
	MH	0.50	0.90	0.64	1.0	1.0	1.0	1.0	1.0	1.0	0.90	1.0	0.95	1.0	0.40	0.57	0.90	1.0	0.95	1.0	1.0	1.0
	MS	0.50	0.90	0.64	1.0	1.0	1.0	1.0	1.0	1.0	0.66	1.0	0.80	1.0	0.20	0.33	0.76	1.0	0.87	1.0	1.0	1.0
	Ray	0.39	0.90	0.54	1.0	1.0	1.0	1.0	1.0	1.0	0.83	1.0	0.91	1.0	0.70	0.82	0.83	1.0	0.90	1.0	1.0	1.0
100 s	n/a	0.18	0.87	0.29	0.21	0.98	0.35	0.21	0.84	0.34	0.47	0.97	0.63	0.92	0.87	0.89	0.98	0.84	0.91	0.97	0.89	0.93
	MH	0.35	0.87	0.50	0.88	0.99	0.93	0.67	0.86	0.75	0.78	0.99	0.87	0.93	0.79	0.85	0.99	0.82	0.90	0.97	0.89	0.93
	MS	0.35	0.87	0.50	0.69	0.99	0.81	0.63	0.86	0.73	0.73	0.99	0.84	0.93	0.80	0.86	0.99	0.84	0.90	0.97	0.89	0.93
	Ray	0.25	0.87	0.38	0.98	0.99	0.98	0.75	0.86	0.80	0.83	0.99	0.90	0.94	0.86	0.90	0.97	0.85	0.91	0.97	0.89	0.93
400 s	n/a	0.84	0.88	0.86	0.95	0.99	0.97	0.95	0.83	0.88	0.91	0.97	0.94	0.97	0.88	0.93	0.98	0.69	0.81	0.99	0.88	0.93
	MH	0.88	0.88	0.88	0.99	0.99	0.99	0.98	0.84	0.90	0.97	0.99	0.98	0.98	0.83	0.90	0.99	0.70	0.82	0.99	0.89	0.94
	MS	0.87	0.88	0.87	0.98	0.99	0.99	0.98	0.84	0.90	0.96	0.99	0.98	0.98	0.84	0.91	0.99	0.68	0.81	0.99	0.89	0.94
	Ray	0.95	0.85	0.90	0.99	0.99	0.99	0.99	0.84	0.91	0.99	0.98	0.99	0.90	0.02	0.04	0.98	0.46	0.63	1.0	0.22	0.36

**Table 2:** Assembly statistics for all assemblers on simulated (10 s, 100 s, 400 s) and real (ERR2017411, ERR2017412) data.

Statistics	Dataset	MEGAHIT	metaSPAdes	Ray
**Synthetic Data**				
number of contigs	10 s	1069	1156	3256
largest contig	10 s	835,563	1,436,250	294,361
avg contig	10 s	31,529.53	29,211.97	10,307.24
n50	10 s	131,416	234,206	31,735
number of contigs	100 s	156,074	210,765	717,512
largest contig	100 s	573,139	190,202	14,995
avg contig	100 s	1936.78	1448.98	189.24
n50	100 s	3051	2732	177
number of contigs	400 s	488,142	901,182	59,663
largest contig	400 s	21,914	13,618	3367
avg contig	400 s	377.24	323.58	149.72
n50	400 s	361	319	138
**Real Data**				
number of contigs	ERR2017411	85,426	165,252	252,974
largest contig	ERR2017411	516,770	394,993	278,191
avg contig	ERR2017411	1606.59	981.96	443.97
n50	ERR2017411	4063	2820	1620
number of contigs	ERR2017412	67,750	141,689	201,038
largest contig	ERR2017412	212,503	264,186	192,118
avg contig	ERR2017412	1360.63	807.96	340.48
n50	ERR2017412	2720	1816	432

**Table 3:** Number of species predicted by each classifiers.

	Kaiju	CLARK	Kraken	MetaCache	MetaPhlAn2	DUDes	GOTTCHA
26,666,674 paired-end reads (10 s) length of 75 bp							
n/a	3553	372	346	50	10	9	10
MEGAHIT	25	10	10	11	5	11	10
MetaSPAdes	31	10	10	15	3	13	10
Ray	36	10	10	12	8	12	10
26,667,004 paired-end reads (100 s) length of 75 bp							
n/a	3659	394	380	176	87	73	84
MEGAHIT	1258	95	125	108	80	71	84
MetaSPAdes	1328	122	131	115	81	72	84
Ray	2109	86	107	101	86	74	84
26,665,698 paired-end reads (400 s) length of 75 bp							
n/a	3707	416	405	426	402	282	390
MEGAHIT	2024	403	394	411	370	284	388
MetaSPAdes	2522	405	396	416	375	277	389
Ray	754	398	392	394	10	188	99
17,853,919 paired-end reads (ERR2017411) length of 90 bp							
n/a	3654	3140	3638	1071	79	29	37
MEGAHIT	2071	1477	1537	718	29	47	25
MetaSPAdes	2618	1782	1867	797	32	33	25
Ray	2679	1630	1731	515	31	40	23
17,793,507 paired-end reads (ERR2017412) length of 90 bp							
n/a	3647	3075	3651	1044	82	48	45
MEGAHIT	1653	1035	1058	611	23	33	26
MetaSPAdes	2312	1387	1423	679	39	42	29
Ray	2192	1203	1297	448	21	21	22

**Table 4:** Pairwise similarity of a method to other methods.

	Kaiju	CLARK	Kraken	MetaCache	MetaPhlAn2	DUDes	GOTTCHA
17,853,919 paired-end reads (ERR2017411) length of 90 bp							
n/a	0.66	0.69	0.66	0.68	0.65	0.82	0.80
MEGAHIT	0.51	0.63	0.62	0.60	0.76	0.81	0.80
MetaSPAdes	0.53	0.65	0.64	0.63	0.73	0.81	0.81
Ray	0.50	0.64	0.62	0.63	0.74	0.81	0.80
17,793,507 paired-end reads (ERR2017412) length of 90 bp							
n/a	0.66	0.69	0.65	0.68	0.71	0.82	0.82
MEGAHIT	0.51	0.63	0.62	0.60	0.76	0.81	0.80
MetaSPAdes	0.53	0.65	0.64	0.63	0.73	0.81	0.81
Ray	0.50	0.64	0.62	0.63	0.74	0.81	0.80

## Abbreviations

The following abbreviations are used in this manuscript:

NGS	Next generation sequencing
SMS	Single-molecule sequencing
GC	Guanine-cytosine
PacBio	Pacific Biosciences
ONT	Oxford Nanopore Technologies
rRNA	Ribosomal ribonucleic acid
WMS	Whole metagenome sequencing
AMR	Antimicrobial resistance
NCBI	National Center for Biotechnology Information
MPI	Message Passing Interface
